# Special Issue: Supplementary Cementitious Materials in Concrete, Part I

**DOI:** 10.3390/ma14092291

**Published:** 2021-04-28

**Authors:** Alessandro P. Fantilli, Daria Jóźwiak-Niedźwiedzka

**Affiliations:** 1 Department of Structural Geotechnical and Building Engineering, Politecnico di Torino-DISEG, Corso Duca degli Abruzzi 24, 10129 Torino, Italy; alessandro.fantilli@polito.it; 2 Department of Experimental Mechanics, Institute of Fundamental Technological Research, Polish Academy of Sciences, Pawińskiego 5B, 02-106 Warsaw, Poland

## 1. Introduction

The environmental impact of the Portland cement production and the large use of cement-based building materials is a growing problem. The substitution strategy, consisting of the partial replacement of Portland cement with supplementary cementitious materials (SCMs), or the more common application of blended cements, is an effective way to improve the sustainability of the construction industries. 

To date, the most common SCM is siliceous fly ash [1], a by-product of coal burning in power plants. However, fly ash, which is used in the production of both cement and concrete, is slowly losing primacy due to the progressive decommissioning of coal-fired power plants. Granulated blast furnace slag [2], generally used to reduce the clinker content, cannot replace fly ash due to its properties and limited availability. On the other hand, fly ash from biomass combustion [3] or natural (pumice, volcanic tuffs) [4] or artificial pozzolans (metakaolin) [5] are increasingly being considered within the building materials industry. 

The issues related to the possibility of extending the range of SCMs, including, for instance, calcareous fly ash [6], wood ash [7], or activated copper tailings [8], are related to their physical and chemical properties, which in turn can enhance some concrete properties (performance strategy). Indeed, due to the use of SCMs, differences in the microstructure are observed in cement and concrete, consisting in the reduction of the total volume of open pores in the hardened cement paste and in the contact zone between paste and aggregate grains. This improves the performances of cement-based composites, especially in terms of durability, or of resistance to an aggressive environment (due to carbonation [9], presence of chloride ions [10], sulphates [11], etc.), by increasing, for instance, water tightness [12]. 

Accordingly, in this Special Issue of Materials, aimed at recognizing the current state of knowledge and development in the use of SCMs within the substitution and performance strategies, the following aspects are investigated:

- Measuring the chemical, physical, and mineralogical properties of SCMs, before and after hydration.
- Defining the amounts and the types of SCMs in accordance with the desired effects on fresh and hardened concrete performances.
- Designing structural elements made with normal and high-performance concretes containing SCMs.
- Assessing the durability and environmental impact of cement-based composites containing SCMs.

Hence, various research issues regarding SCMs are herein considered and described in detail through both research papers and state-of-the art reviews. The articles featured in this Special Issue cover the aspects of design, testing, and application of various types of supplementary cementitious materials in concrete. The results of the research, conducted by over 45 international universities and scientific centers, prove the great interest in the SCM topic. In fact, they foster the introduction of new and more environmentally friendly construction materials, with improved physical and mechanical properties, to be used in building engineering.

## 2. Short Description of the Articles Presented in This Special Issue

The issues of the original research papers and stat-of-the art reviews published in this Special Issue of Materials, and coming from over 45 international universities and scientific centres, can be divided as follows:

- permeability and diffusivity, which are directly related to the quality of concrete and to the durability in various aggressive environments [13,14,15,16,17];
- properties of modified cement matrix composites reinforced with fibers [14,18,19,20];
- application of new types of supplementary cementitious materials [13,16,21,22,23,24,25,26,27,28];
- characterization of various types of modified cement based materials [18,21,23,25,26,27,28,29,30,31].

In the study presented by Andrade et al. [13] a common clay–bentonite was used as concrete additive. The Authors not only analysed the influence of such clay on the mechanical properties of concrete, but also its chemical resistance to sulphates, carbonation and chlorides. They revealed that in the bentonite-bearing material, the resistance to carbonation can be lower than in reference plain Portland cement. 

Ahmed et al. [32] showed detailed characteristic of the high-performance self-consolidating concrete incorporating waste mineral materials (i.e., micro-silica and fly ash). They analysed fresh and hardened properties of concrete, and introduced a multi-parameter analytical approach to identify the optimum concrete mixture in terms of cost, workability, strength, and durability. 

In the paper presented by Ali et al. [14] the individual and combined incorporation of steel fiber and micro-silica in high-strength concrete were investigated. The tailoring of concrete were performed according to the results of mechanical and permeability tests. By varying the fiber dosage, a mixed effects on the permeability of concrete (water absorption and chloride ion penetration) occurs. However, the presence of micro-silica minimalized the negative effects of high fiber dosage on the properties of concrete.

Research performed by Vu et al. [21] concerned the introduction of new drywall wood-based particleboard as an alternative to gypsum board. More precisely, the use of wood particles in combination with steatite powder and Portland cement was investigated. Both screw withdrawal resistance and bending properties were improved with respect to gypsum board having a similar density. Authors also revealed that wood-cement-steatite powder particleboard could be classified as a quasi-non-combustible material.

Han et al. [31] investigated the application of porous feldspar to reduce the use of cement and sand in the heat storage concrete layer. The mechanical and chemical activation methods were used to compensate the reduction of strength, due to the lower content of cement. With respect to a reference cement mortar, the compressive strength was approximately twice when chemical activation was performed after reducing the cement content by 5% and replacing the sand with porous feldspar. In a large-scale model experiment, the heat storage layer containing the porous feldspar exhibited better thermal properties than those of heat storage layers made with ordinary cement mortar. 

The study presented by Yang et al. [29] was aimed at introducing a precise strength evaluation technique. The apparent activation energy of ground granulated blast furnace slag (GGBFS) was calculated through several experiments and used to set up a prediction model. The latter, based on the thermodynamic reactivity of GGBFS within a concrete system cured at different temperature, was able to estimate the compressive strength of GGBFS concrete in accordance to the experimental results.

Kalinowska-Wichrowska et al. [22] presented the results of using recycled cement mortar, obtained from old concrete, as a supplementary cementitious material. Authors showed that by means of a thermal treatment of concrete rubble, a high-quality fine fraction can be obtained. In particular the fine material has pozzolanic properties and can be used as a partial cement replacement in new mortar and concrete.

The parameters affecting the fibre pullout capacity and strain-hardening behavior of fibre-reinforced alkali-activated cement-based composites were investigated by Lee et al. [18]. They used fly ash as a common aluminosilicate source in alkali-activated cementitious composite, whose compressive and flexural strengths were analyzed in addition to the strain-hardening behavior. In particular, the composite critical energy release rate was determined with a nanoindentation approach.

Fantilli et al. [19] investigated the use of ultra-high performance fibre-reinforced cementitious composites (UHP-FRCC), made with various replacement ratio of cement with fly ash, as a reinforcement material of existing concrete columns. Relationships between the size of the UHP-FRCC jacket, the percentage of cement replaced by fly ash, and the strength of the columns were measured and analyzed by means of the eco-mechanical approach. They found that replacement of approximately half of cement with fly ash, and a suitable thickness of the ultra-high performance fibre-reinforced cementitious composites jacket, could ensure the lowest environmental impact without lowering the mechanical properties.

In the study presented by Ishak et al. [23], the influence of the high temperature (from 200 °C to 800 °C) on the fly ash geopolymer concrete incorporating bamboo ash was investigated. When 5% bamboo ash is added to fly ash, geopolymers exhibited more than 50% improvement in residual strength. Moreover, bamboo origin fly ash could be one of the alternatives to fly ash when geopolymer concrete is exposed to high temperature. 

Research performed by Monfardini et al. [20] was focused on the flexural behavior of structural elements made with alkali-activated concrete containing class F fly ash, and reinforced with conventional steel rebars in combination with fibers randomly distributed within the concrete matrix. The benefical effects of the hybrid reinforcement was measured through experimental analyses performed on full-scale strutcure at ultimate and serviceability limit states.

Hager et al. [15] analyzed the influence of the binder type (Portland cement and slag cement) on the mechanical and transport properties of heated concrete. The compressive and tensile strength, as well as the static modulus of elasticity and permeability, were measured after the exposure to elevated temperatures (from 200 °C to 1000 °C). The damage of concrete and crack growth due to high temperatures were quantified in accordance with the variation of the static modulus of elasticity. Test results clearly showed the existence of an exponential increment of permeability with damage for both the types of cement.

Vukićević et al. [24] proposed the use of alternative waste materials and hydraulic binders for the soft soil stabilization. High plasticity clay stabilization using fly ash, as well as engineering properties of ash and ash-slag mixtures, were investigated. Test results showed the positive effects of clay stabilization using fly ash, in terms of increasing strength and stiffness and reducing expansivity. As the mechanical properties of fly ash and ash-slag mixtures were comparable with those of sands, they can be used as sustainable fill materials for embankments.

Glinicki et al. [25] investigated the influence of fluidized bed combustion fly ash on the phase composition and microstructure of cement paste. They observed a significant changes in portlandite content and only moderate changes in the content of ettringite, especially when a quantitative evaluation of the phase composition, as a function of fluidized bed fly ash content, was performed.

The aim of the research presented by Vu et al. [26] concerned the use of biomass wood ash as a partial replacement of cement material in wood-cement particleboards. Test results indicated that water demand increased with the increasing of the ash content, and the mechanical properties decreased slightly with an increase of the ash content. The heat capacity increased with the wood ash content as well. The replacement of cement to an extent of approximately 30% by weight was found to give the optimum result. 

Woyciechowski et al. [16] presented a new self-terminatin model of carbonation, where the content of calcareous fly ash was taken into consideration as binder component. Also, the idea of developing models for various concrete compositions, as a tool for designing concrete cover thickness of reinforced elements, was proposed.

Fantilli et al. [30] investigated the structural behavior of reinforced concrete elements made with fly ash substitution. A new procedure was introduced with the aim of fulfilling a new limit state of sustainability, in accordance with the serviceability and ultimate limit states currently required by building codes. The proposed approach showed that the CO_2_ emission of a reinforced concrete beam was not a monotonic function of the substitution rate of cement with fly ash. On the contrary, there were favorable values of such substitution rates.

García-Vera et al. [17] presented a research on the effect produced by an aggressive environment (containing sulphuric acid solution) on mortars containing different percentages of a crystalline admixture. After a sulphuric acid exposure, mortars made with crystalline admixtures showed higher compressive strength than the reference mortars, besides exhibiting lower mass loss. However, the crystalline admixture did not produce any significant effect on the capillary water absorption coefficient. Whereas, in the short term analysis made in a nonaggressive environment, the crystalline admixture did not have a significant effect neither on the compressive strength and on the capillary water absorption coefficient, nor on the ultrasonic pulse velocity.

The state-of-the-art review presented by Jaskulski et al. [27] concerned various aspects of calcined clays application as a supplementary cementitious material. In more than 200 recent research papers, the authors discussed in detail the idea of replacing Portland cement with large amounts of calcined clay. 

Finally, Nicoara et al. [28] reviewed a series of papers regarding the use of end-of-life materials as SCMs in the concrete industry. Ordinary Portland Cement can be effectively substituted by several industrial end-of-life products that contain calcareous, siliceous and aluminous materials, as well as by natural pozzolanic materials like sugarcane bagasse ash, palm oil fuel ash, rice husk ash, mine tailings, marble dust, and construction and demolition debris. Authors revealed that the application of the above-mentioned waste materials as SCMs would decrease the amount of cement used in the production of concrete, and reduce the carbon emissions associated with cement production.

## 3. Conclusions

The application of supplementary cementitious materials in cement-based composites was an appropriate Special Issue choice, as evidenced by the wide number of research papers published on this subject. They primarily concerned the characterization of new types of SCMs and their possible use in cement-based composites. The properties of matrixes containing SCMs could be further improved by the presence of a fiber reinforcement. Moreover, a better durability in various aggressive environments was also observed. Thus, new building materials containing SCMs can be effectively tailored with the aim of substituting traditional virgin raw materials and to increase their performances, espcially in terms of reducing CO_2_ and NO_x_ emissions.
